# Use of preferred contraceptive method among young adults in Texas and California: A comparison by state and insurance coverage

**DOI:** 10.1371/journal.pone.0290726

**Published:** 2023-08-31

**Authors:** Kristine Hopkins, Jennifer Yarger, Irene Rossetto, Audrey Sanchez, Elisa Brown, Sarah Elmes, Thaddeus Mantaro, Kari White, Cynthia C. Harper

**Affiliations:** 1 Population Research Center, The University of Texas at Austin, Austin, Texas, United States of America; 2 Department of Epidemiology and Biostatistics, University of California, San Francisco, San Francisco, California, United States of America; 3 Philip R. Lee Institute for Health Policy Studies, University of California, San Francisco, San Francisco, California, United States of America; 4 Department of Obstetrics and Gynecology, Texas Tech University Health Sciences Center, Odessa, Texas, United States of America; 5 Department of Obstetrics, Gynecology and Reproductive Sciences, University of California, San Francisco, San Francisco, California, United States of America; 6 Health Services and Promotion, Dallas College, Dallas, Texas, United States of America; 7 Steve Hicks School of Social Work, The University of Texas at Austin, Austin, Texas, United States of America; Sindh Social Protection Authority, PAKISTAN

## Abstract

**Background:**

Young people’s ability to use their preferred contraceptive method is an indicator of reproductive autonomy and healthcare access. State policies can hinder or facilitate access to a preferred contraceptive method.

**Objective:**

This study compared use of preferred contraceptive method in Texas and California, states with contrasting health policy contexts that impact health insurance coverage and access to subsidized family planning services.

**Methods:**

We used baseline survey data from an ongoing cluster randomized controlled trial of sexually active students, assigned female at birth, ages 18–25, at 29 community colleges in Texas and California (N = 1,974). We described contraceptive preferences and use, as well as reasons for nonuse of a preferred method. We conducted multivariable-adjusted mixed-effects logistic regression analyses for clustered data, and then calculated the predicted probability of using a preferred contraceptive method in Texas and California by insurance status.

**Results:**

More Texas participants were uninsured than Californians (30% vs. 8%, p<0.001). Thirty-six percent of Texas participants were using their preferred contraceptive method compared to 51% of Californians. After multivariable adjustments, Texas participants had lower odds of using their preferred method (adjusted odds ratio = 0.62, 95% confidence interval = 0.48–0.81) compared to those in California. Texas participants in all insurance categories had a lower predicted probability of preferred method use compared to California participants. In Texas, we found a 12.1 percentage-point difference in the predicted probability of preferred method use between the uninsured (27.5%) and insured (39.6%) (p<0.001). Texans reported financial barriers to using their preferred method more often than Californians (36.7% vs. 19.2%, p<0.001) as did the uninsured compared to the insured (50.9% vs. 18.7%, p<0.001).

**Conclusion:**

These findings present new evidence that state of residence plays an important role in young people’s ability to realize their contraceptive preference. Young people in Texas, with lower insurance coverage and more limited access to safety net programs for contraceptive care than in California, have lower use of preferred contraception. It has become urgent in states with abortion bans to support young people’s access to their preferred methods.

## Introduction

The ability for young people to use their preferred contraceptive method is an indicator of health care access and reproductive autonomy [1]. However, a growing literature demonstrates that current contraceptive method use often does not reflect method preferences. Several recent studies have found a mismatch between the methods that people use and the methods they want to use, with many desiring to use a more effective method [2–6]. Cost barriers are frequently cited as reasons for unfulfilled preferences [2–4, 7]. Analyses of the 2015–2017 National Survey of Family Growth found that 22% of women at risk of an unplanned pregnancy would have preferred a different contraceptive method if cost were not a factor [8] and 39% of nonusers of contraception would start using a method if not for the cost [9]. However, results are mixed on the association between insurance coverage and preference-use mismatch. Some studies found that uninsured people were less likely to be using their preferred method [4, 5], while others found no association [3, 6].

State-level policies, including whether a state has expanded Medicaid, may facilitate access to contraception by increasing insurance coverage and reducing cost and other barriers [10]. Thirty-eight states, including California, have expanded full-benefit Medicaid coverage [11]. California’s Medicaid program, Medi-Cal, covers all U.S. citizens, permanent residents, and legal residents who live in households with incomes up to 138% of the federal poverty level (FPL) [12]. California’s program also covers young adults under age 26 [13], regardless of immigration status. In contrast, twelve states, including Texas, have not expanded full Medicaid. Texas extends full Medicaid coverage only to U.S. citizens and legal immigrants who are parents/guardians of dependent children and have incomes up to 16% FPL [14].

While both states have implemented policies to provide contraception to people who do not qualify for full-benefit Medicaid but who meet other eligibility criteria [15] such as lower incomes or for those who cannot use their insurance because of confidentiality concerns [16], California’s program covers far more people. California’s Family Planning, Access, Care and Treatment (Family PACT) program provides comprehensive family planning services at no cost to all California residents of childbearing age who have incomes of up to 200% FPL and no other source of insurance coverage for family planning services or who have confidentiality concerns [17].

In contrast, Texas’s program, Healthy Texas Women, covers U.S. citizens and qualified legal immigrants who can get pregnant with incomes of up to 204% FPL [18]. Rather than implement policies to maintain or expand coverage, Texas has implemented a series of restrictive state policies that have excluded certain providers from participating in family planning programs [19, 20], which led to a large number of family planning clinic closures [21–23].

Given differing state policy environments affecting insurance coverage, it is not surprising that Texas and California have large differences in the percentage of young adults who are uninsured. In Texas, 30% of 19–25 year old young adults were uninsured in 2019 compared to 12% in California [24]. However, even in states with more expansive health coverage policies for both the insured and uninsured, young adults, including students, may still have cost concerns. A study of community college students aged 18–25 in California and Oregon found that nearly half of students were concerned about the cost of contraception, and that the uninsured had the highest cost concerns [25].

In this study, we examined the impact of no insurance coverage on use of a preferred method of contraception in Texas and California. Specifically, we compare mismatch between contraceptive preference and use between young people in these states with contrasting contraceptive policy and service environments. We focus on young people attending community college because more students from lower income families attend two year institutions than attend four year institutions [26], and this overwhelming difference contributes to structural inequities that limit access to health insurance [27].

## Methods and materials

We used baseline survey data from an ongoing cluster randomized controlled trial of first-year community college students, assigned female at birth, ages 18–25, who had vaginal sex with a male partner in the last year, and were not currently pregnant or wanting to become pregnant in the next year [28]. We enrolled students attending 29 community college sites in Texas and California from April 2018 through May 2023. Of these 29 colleges, two were located in rural (nonmetropolitan) counties and the remainder in metropolitan counties– 15 large central metro, two large fringe metro, nine medium metro, and one small metro [29].

Pre-pandemic, we recruited and enrolled students in person, which we completed at 17 colleges. After March 2020, we shifted to remote recruitment and enrollment. We conducted exclusively remote recruitment and enrollment at four colleges. At the remaining eight colleges, we resumed in-person recruitment at a later date, ranging from August 2021 through April 2023, and continued with remote enrollment. Throughout the study, in both California and Texas, college staff sent emails to students and posted study information on college course content management systems inviting students to participate. In addition, in Texas, where students’ names and email addresses were available upon request, the study team emailed first-year female students. Students were given a written consent form and provided electronic consent to participate. Participants then completed baseline surveys covering sociodemographic characteristics, health insurance status, and reproductive health history, including current and preferred contraceptive methods. Study participants received remuneration of a $50 electronic gift card following study enrollment.

This study was approved by the Institutional Review Boards (IRBs) at the University of California, San Francisco (#17–23183) and The University of Texas at Austin (#2019010078); participating college sites either approved the study with their IRB (7 sites) or used the corresponding state university’s IRB approval (22 sites).

### Measures

To measure current contraceptive method, participants selected their method or methods from a list. For those who selected more than one method, we used the method that participants identified as their “main method” of contraception. We categorized methods into the following: oral contraceptive pill, transdermal patch or vaginal ring, intrauterine device (IUD) or sub-dermal implant, injectable, condom, fertility awareness, emergency contraception or other, withdrawal, and no method.

To measure preferred contraceptive method, participants were asked, “If you could use any birth control method you wanted, what method would you use?” Response options included “I am using the method that I want,” and the same list of contraceptive methods as for current contraceptive methods, with the addition of male and female sterilization and “don’t know.” For participants who selected multiple preferred contraceptive methods, we selected as preferred method the one that matched their current main method (n = 141). For the remaining participants (n = 194), we did not want to prioritize any particular method characteristic when assigning preferred method because evidence indicates that people choose contraceptive methods for a range of reasons [30]. Therefore, we randomly assigned a preferred method from the list of methods these participants selected.

The primary study outcome is whether participants reported that they were using their preferred method of contraception. Students whose current contraceptive method was the same as their preferred method were coded as using their preferred method.

We also explored challenges in using a preferred contraceptive method. Participants who were not using their preferred method were asked why not and could choose multiple responses from a list of reasons as well as write in other reasons. We grouped 1,452 responses from the 1,002 participants who gave a reason or reasons for not using their preferred method into several categories. Information/availability barriers included “I don’t know where to get it,” “It’s too much hassle to get it,” “My doctor/clinic doesn’t offer it,” and “My doctor advised against it.” Parents/partner barriers included “My parents don’t know that I’m sexually active” and “My partner doesn’t want me to use it.” Financial barriers were “I can’t afford it” and “My insurance doesn’t cover it.” Other reasons were fear of side effects; not needing a method, which included “I’m not sexually active” and mentions of not wanting or needing a method; those who stated that they had an appointment to get the method soon; and other reasons (e.g., it feels better without a condom).

Our independent variables include state, Texas or California, and health insurance status, uninsured, insured (private, Medicaid), and did not know insurance status.

We included sociodemographic covariates that have been associated with contraceptive preferences in previous research [3, 4, 6]. Covariates were age, categorized into adolescents (18–19 year-olds) and young adults (20–25 year-olds), has a child or children (yes, no), self-reported race and ethnicity (Hispanic, White non-Hispanic, Asian/Pacific Islander non-Hispanic, Black non-Hispanic, American Indian/Other/Multi-racial non-Hispanic; for brevity, Latinx, White, Asian/Pacific Islander, Black, American Indian/Multi-racial/other), and language spoken at home (English, language other than English).

### Statistical analysis

From the total sample of 2,084, we excluded observations for those who did not state a preference (i.e., answered “don’t know”; n = 94), were missing whether they were using their preferred method (n = 5), had missing data on the sociodemographic covariates (n = 8), or were missing on all these variables (n = 3), for a final analytical sample of 1,974. Given that study participants are clustered within colleges, in the descriptive statistical analysis, we used univariate logistic regression models with cluster robust standard errors to test for differences between the state samples and between those who were and were not using their preferred method. Finally, we conducted multivariable-adjusted mixed-effects logistic regression analyses for clustered data to assess participant likelihood of using their preferred contraceptive method. We estimated a main effects model with our two independent variables of state and insurance status. Next, given the much higher rates of uninsured in Texas than in California, we modeled the interaction between state and insurance status. Finally, in the full model, we added sociodemographic controls: age, race/ethnicity, has a child, and language spoken at home. Using the full model, we calculated margins to illustrate the predicted probability of using a preferred contraceptive method in Texas and California and by insurance status in each state. All analyses were conducted in Stata 17 (College Station, TX).

## Results

Over 29% of Texas participants were uninsured compared to 7.7% of California participants (p<0.001) (Table 1). The majority of participants in both states were 18–19 years old with no children. The Texas sample was slightly older, with 27.2% who were 20–25 years old at enrollment compared to 15.2% in California (p<0.01); more Texas participants also had a child (8.8% vs. 3.2%, p<0.001). The state samples were racially and ethnically diverse, with the Texas sample having a larger percentage of Latinx participants (71.5% in Texas vs. 51.6% in California; p<0.05) and Black participants (10.2% vs. 4.2%, p<0.05). The study samples were largely reflective of the racial and ethnic composition of community college student populations in each state [31, 32]. More Texas participants reported speaking a language other than English at home (58.1 vs. 46.4%), though the difference was not statistically significant.

**Table 1:** Participant characteristics by state (N = 1,974).

	State			
	Texas (n = 558)		California N = 1,416)	
**Insurance status**				
Uninsured	164	29.4***	109	7.7
Insured (ref)	332	59.5	1,153	81.4
Don’t know status	62	11.1	154	10.9
**Age**				
18–19 (ref)	406	72.8	1,201	84.8
20–25	152	27.2**	215	15.2
**Has child(ren)**				
No	509	91.2	1,371	96.8
Yes (ref)	49	8.8***	45	3.2
**Race/ethnicity**				
Hispanic/Latinx	399	71.5*	730	51.6
White (ref)	70	12.5	342	24.2
Black	57	10.2*	60	4.2
Asian/Pacific Islander	20	3.6	176	12.4
Amer. Indian/ Multi–racial/other	12	2.2	108	7.6
**Language spoken at home**				
English	234	41.9	759	53.6
Language other than English (ref)	324	58.1	657	46.4

Note: * p <05

** p < .01

*** p <0.001

Univariate logistic regression models with cluster robust standard errors compared California to Texas.

**Fig 1:**
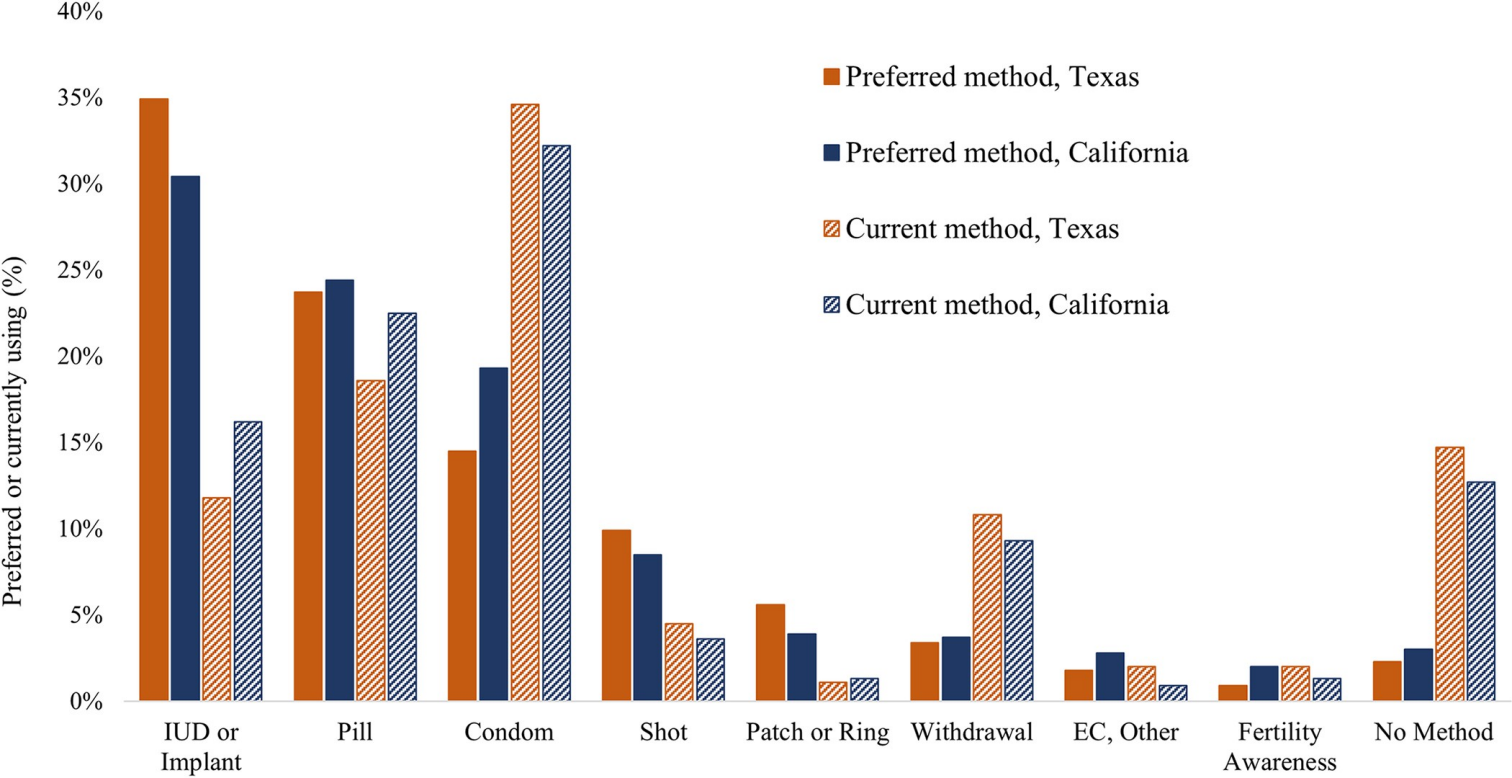
Contraceptive preferences and use in Texas and California (N = 1,974). Note: No students were using sterilization at baseline; preference for sterilization was 3.0% in Texas and 2.0% in California.

Fig 1 shows contraceptive preferences and use for Texas and California. In both states, preference was highest for the IUD or implant (34.9% in Texas, 30.4% in California), while these methods were used by 11.8% in Texas and 16.2% in California. Oral contraceptive pills were the second-most preferred method in both states (23.7% in Texas and 24.4% in California) and were used by 18.6% in Texas, 22.5% in California. Although not as many participants reported a preference for condoms (14.5% in Texas, 19.3% in California), condoms were used by the most participants in both states (34.6% in Texas, 32.2% in California). Just 2.3% of participants in Texas and 3.0% in California stated a preference for using no method, while 14.7% of Texans and 12.7% of Californians reported using no method.

A lower percentage of Texas participants reported using their preferred method of contraception than California participants (36.2% vs. 51.3%; p<0.001) (Table 2). Uninsured participants (36.3%) were less likely to report using their preferred method of contraception compared to those who were insured (49.5%, p<0.001). Participants who did not know their insurance status had a lower percentage of using their preferred method (44.0%) compared to the insured, but the difference was not statistically significant.

**Table 2:** Percent using preferred contraceptive method, by state and insurance status (N = 1,974).

	Using preferred contraceptive method			
	Yes		No	
	N	%	n	%
**State**				
Texas	202***	36.2	356	63.8
California (ref)	727	51.3	689	48.7
**Insurance status**				
Uninsured	99	36.3	174***	63.7
Insured (ref)	735	49.5	750	50.5
Don’t know status	95	44.0	121	56.0

Note: *** p <0.001

Univariate logistic regression models with cluster robust standard errors to compare those who realized their contraceptive preference to those who did not.

The primary reasons that participants in Texas and California reported for not using their preferred method were information/availability barriers (48.1% and 49.6%), and parent/partner barriers (36.0% and 36.6%) (Table 3). Financial barriers were more frequently reported by participants in Texas than in California (36.9% vs. 19.2%), among the uninsured (50.9%) and those who did not know their insurance status (27.2%) compared to the insured. In addition, fewer Texas participants reported not needing a method compared to those in California (8.7% vs. 12.1%). Fewer of the uninsured reported fear of side effects as a barrier to their preferred method use compared to the insured (5.2% vs. 12.3%).

**Table 3:** Reasons for not using preferred contraceptive method, by state and insurance status (%).

	State		Insurance Status		
Reason	Texas	California	Uninsured	Insured	Don’t know
Information/availability barriers	48.1	49.6	46.2	49.5	50.9
Parent/partner barriers	36.0	36.6	37.0	36.1	37.7
Financial barriers	36.9***	19.2	50.9***	18.9	27.2*
No need	8.7*	12.7	9.3	11.5	13.2
Fear of side effects	8.7	12.1	5.2*	12.3	11.4
I have an appointment scheduled to get it soon	6.3	6.6	6.9	6.7	4.4
Other (e.g., it feels better without a condom)	4.0	6.1	4.6	5.9	2.6

***p<0.001

*p<0.05

Note. Multiple responses allowed; percentage of 1,452 responses received from 1,002 total participants; responses missing for 43 participants. Univariate logistic regression models with cluster robust standard errors were used to test for differences by state and insurance status. The question was not asked of those who were using a method but wanted to use no method (n = 30).

In multivariable-adjusted mixed-effects logistic regression analyses, participants from Texas had lower odds of using their preferred method (adjusted odds ratio [aOR] = 0.58, 95% confidence interval [CI] = 0.41–0.82) and the uninsured had lower odds compared to the insured (aOR = 0.71, 95% CI = 0.53–0.95) (Table 4, Model 1). In Model 2, which included an interaction term between state and insurance status, we found that uninsured Texas participants had 68% lower odds of using their preferred method than insured California participants. Finally, the relationships between use of preferred methods and state and insurance persisted after inclusion of sociodemographic controls (Model 3).

**Table 4:** Using preferred contraceptive method: Multivariable-adjusted mixed-effects logistic regression analyses (n = 1,974).

	Model 1: Main effects			Model 2: Main effects + interaction			Model 3: Main effects + interaction + sociodemographic controls^§^		
	aOR	95% CI	p-value	aOR	95% CI	p-value	aOR	95% CI	p-value
**State**									
Texas	0.58	(0.41–0.82)	0.002	0.65	(0.47–0.90)	0.009	0.62	(0.48–0.81)	<0.001
California (ref)	1.00			1.00			1.00		
**Insurance status**									
Uninsured	0.71	(0.53–0.95)	0.021	1.02	(0.64–1.62)	0.942	1.09	(0.68–1.75)	0.713
Insured (ref)	1.00			1.00					
Don’t know	0.83	(0.65–1.06)	0.131	0.81	(0.62–1.07)	0.140	0.84	(0.64–1.10)	0.207
State*Insurance interaction									
Texas*Uninsured				0.48	(0.29–0.81)	0.006	0.51	(0.30–0.87)	0.013
Texas*Don’t know				1.03	(0.56–1.88)	0.923	1.11	(0.62–2.00)	0.725
Constant	1.11	(0.91–1.34)	0.308	1.08	(0.89–1.31)	0.454	1.47	(1.17–1.84)	0.001

Note. aOR = adjusted odds ratio estimated from mixed-effects logistic regression models; CI = confidence interval.

^§^ Sociodemographic controls are age, race/ethnicity, language spoken at home, and has child(ren).

**Fig 2:**
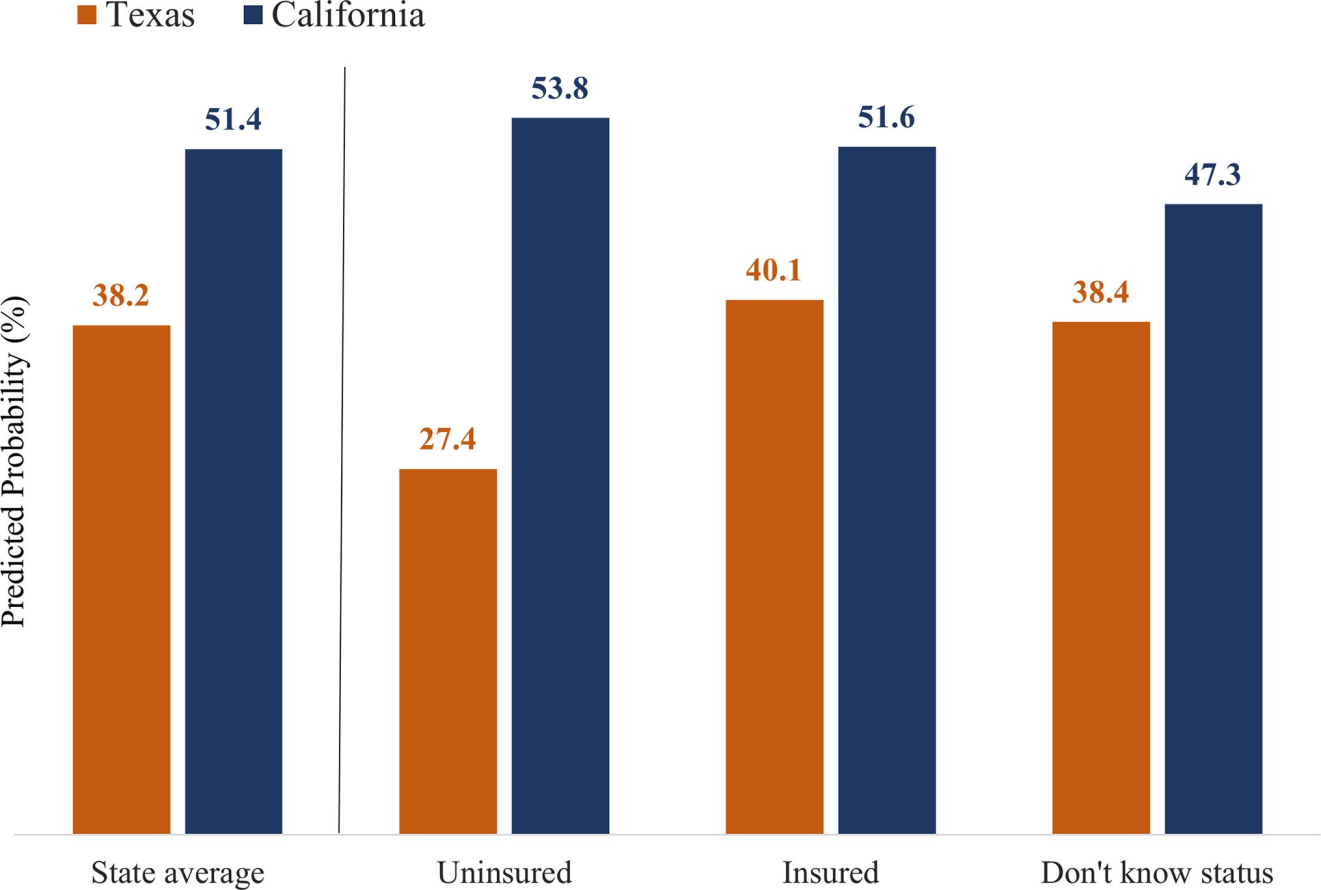
Predicted proportions of using preferred contraceptive method by state and insurance status. Note. Predictions based on Model 3 reported in Table 4.

Fig 2 displays the predicted probability of participants using their preferred method overall in each state and by insurance status, based on the estimates from Model 3 in Table 4. Overall, 38.2% of the Texas participants were predicted to be using their preferred method, compared to 51.4% of California participants (Fig 2). Texas participants in all insurance categories had a lower predicted probability of preferred method use compared to California participants. In Texas, we found a 12.7 percentage-point difference in the predicted probability of preferred method use between the uninsured (27.4%) and insured (40.1%) (p<0.001). The difference between the insured and those who did not know their status (38.3%) in Texas was not significant (p = 0.779). Likewise, the probability of using a preferred method was not significant between the uninsured (53.8%) and insured (51.6%) in California (p = 0.713) or between the insured and those who did not know their status (47.3%, p = 0.206).

## Discussion

We found that about one-third of student participants in Texas and one-half in California were using their preferred method of contraception. Moreover, after controlling for sociodemographic characteristics, we found that fewer uninsured Texas participants were predicted to be using their preferred method compared to insured Texans and no differences by insurance status in California.

These findings present new evidence that state of residence plays an important role in young people’s ability to realize their contraceptive preference, magnified by differences in insurance coverage, as well as through differences in state programs that provide contraceptive coverage for the uninsured. Young people residing in California, which has expanded Medicaid to provide insurance coverage for a broad swath of its low-income population, were more likely to realize their contraceptive preferences compared to young people in Texas. Moreover, we did not find a difference in the realization of contraceptive preference between the uninsured and insured in California, suggesting that California’s strong family planning safety net is accomplishing its goals of providing more widespread contraceptive coverage for the uninsured. In other words, we found higher preferred method use and no difference across insurance status for young people in California thanks to that state’s supportive policies and programs.

The more restrictive healthcare policy environment in Texas, including budget cuts to publicly funded services and restrictions on who can provide those services [19–22], all likely play a role in Texas students’ limited use of their preferred contraceptive method compared to students in California. In addition, Texas’s Medicaid family planning waiver program, which does not extend services to undocumented immigrants, may also play a role in the ability of young Texans in this study to use their preferred method [33], though we are unable to confirm this with our data because, due to its sensitivity, we did not ask about immigration status.

Regardless of where they lived or whether they had insurance, about half of all participants said they were not using their preferred method because of information or availability barriers. Not finding a state difference is somewhat surprising, given that Texas has a long history of abstinence-only or no sex education in schools [34] and California has an equally long history of mandated comprehensive sex education in secondary schools, including a requirement to provide information about local sexual health service providers [35].

Similar to other studies that compared contraceptive use and preferences [2–6], we found that many young adults in community college in this study had unfulfilled preferences for contraception. Our estimates are higher than in studies with populations that included older adults [2, 3], and may be due, at least in part, to confidentiality concerns that young people face [16]. Indeed, over one-third of students in this study noted that the reason they were not using their preferred method of contraception was because they did not want their parents to know that they were sexually active. This is consistent with previous research which found that college students who have less frequent conversations with their mothers about sex are less likely to intend to use reproductive health services [36]. Also similar to other studies, we found that young people want to use more effective methods, such as the IUD, implant or injectable, that often require a clinic visit [4]. Instead, they are relying on methods that may be more convenient and accessible for them (e.g, withdrawal) or can be paid for out of pocket (e.g., condoms).

### Limitations

This study focuses on a sample of community college students, and is representative of that population, but not of all 18 to 25 year-olds in Texas and California. Moreover, the California sample is larger than the Texas sample, and it is possible that if we had a larger Texas sample, we may have found greater differences in use of contraceptive preferences between the states. However, these results are indicative of significant gaps in contraceptive access for young adult community college students in Texas and California. In addition, we did not collect information about immigration status, which means that we can only hypothesize about the potential impact of policies that expand or restrict access to undocumented immigrants on preferred method use.

## Conclusion

These findings present compelling evidence that state of residence, via policies and programs that affect access to services, plays a role in young people’s ability to realize their contraceptive preference. Young people in Texas, with lower insurance coverage and more limited access to safety net programs for contraceptive care than in California, have lower use of preferred contraception. It is critical to expand access to preferred contraception in all states, but it has become urgent in states with abortion bans, such as Texas, to support young people’s access to their preferred methods.
